# Primary Leptomeningeal Oligodendroglioma, IDH-Mutant, 1p/19q-Codeleted

**DOI:** 10.3389/fneur.2018.00700

**Published:** 2018-08-27

**Authors:** Leomar Y. Ballester, Erin Dunbar, Nandita Guha-Thakurta, John W. Henson, Howard Chandler, Jeremiah Watkins, Gregory N. Fuller

**Affiliations:** ^1^Department of Pathology and Laboratory Medicine, Houston, TX, United States; ^2^Neurosurgery, University of Texas Health Science Center at Houston, Houston, TX, United States; ^3^Piedmont Brain Tumor Center, Piedmont Cancer, Atlanta, GA, United States; ^4^Department of Diagnostic Radiology, University of Texas MD Anderson Cancer Center, Houston, TX, United States; ^5^Department of Pathology, University of Texas MD Anderson Cancer Center, Houston, TX, United States

**Keywords:** leptomeningeal oligodendroglioma, IDH1, 1p/19q-codeletion, diffuse glioma, CIC, ATRX, FUBP1

## Abstract

We present a case of a 43-year-old woman with a history of headaches and blurry vision. Ophthalmologic examination identified papilledema. MR imaging demonstrated a right parietal region mass with patchy areas of contrast enhancement and focal calcifications. Intraoperative examination and exploration revealed an extra-axial mass with no apparent parenchymal involvement. Microscopic examination revealed solid sheets of tumor cells with clear cell cytologic features and no discernable intra-parenchymal tumor component. Molecular studies demonstrated the presence of *IDH1 IDH1* c.395G>A p.R132H and *CIC* c.601C>T p.R281W mutations and 1p/19q codeletion. The radiographic features, gross appearance, and microscopic and molecular characteristics of the mass support the diagnosis of primary leptomeningeal oligodendroglioma, IDH-mutant, 1p/19-codeleted. This case represents one of a very few reported instances of molecularly-defined solitary, primary, intracranial oligodendroglioma, without definitive involvement of the brain parenchyma.

## Clinical presentation and imaging results

**Figure 1:**
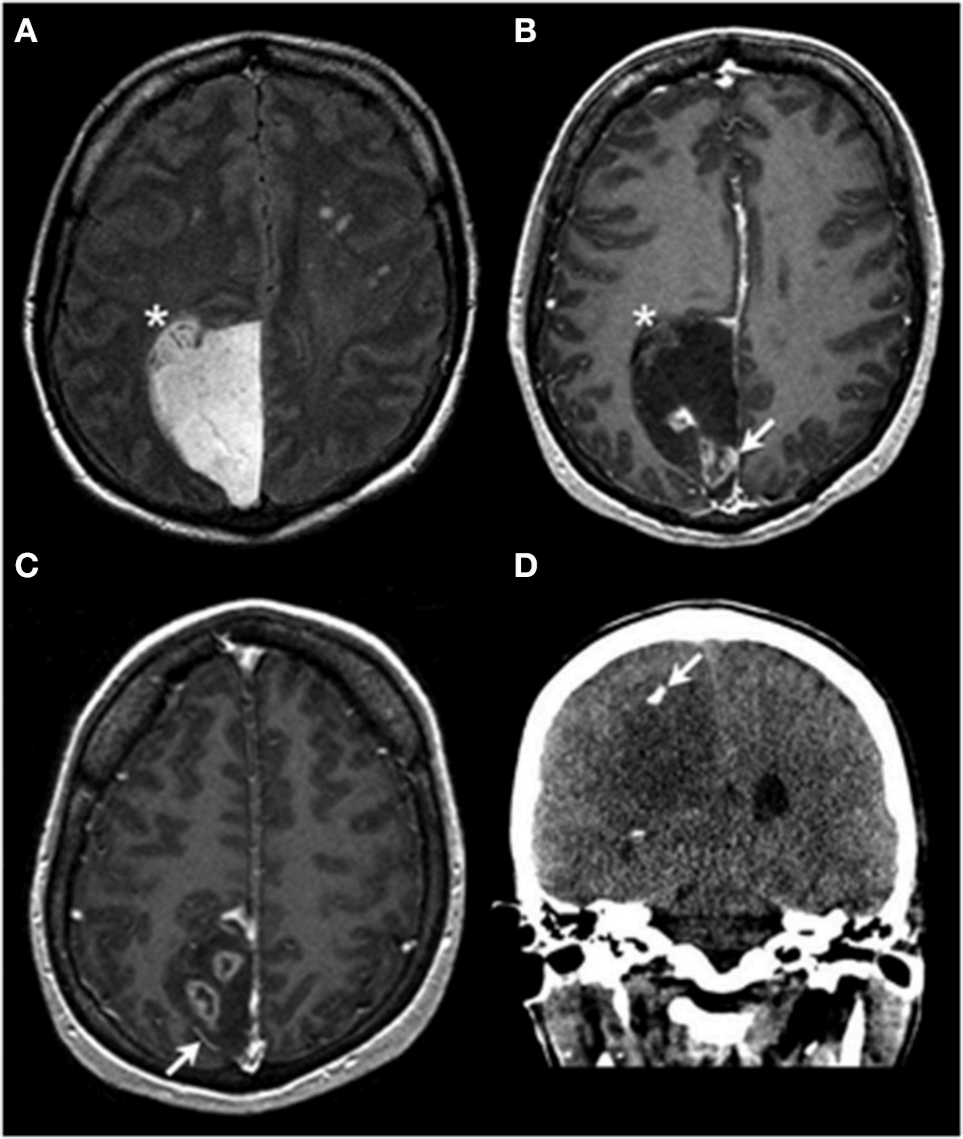
Radiologic findings. Axial T2-weighted FLAIR imaging **(A)** demonstrates an extra-axial hyperintense mass centered within the right paramedian parietal region with a focus that appears to be inseparable from the adjacent cortex (asterisk in **A,B**). On the axial T1 post contrast images **(B,C)** the lesion is predominantly nonenhancing and hypointense, with areas of heterogeneous enhancement (arrow in **B**). Also noted is lateral displacement of a cortical vessel (arrow in **C**), suggestive of an extra-axial location of the mass. Coronal non-contrast CT imaging demonstrates a focus of coarse calcification (arrow in **D**) in the hypoattenuating mass.

A 43-year-old woman presented with several months of blurry vision and headaches. Ophthalmologic examination revealed papilledema. She had no personal or family history of malignancy, but had lived near the Chernobyl, Ukraine nuclear disaster site from birth until her late 20s. Brain magnetic resonance imaging (MRI) demonstrated a right paramedian parietal region mass, ~5 × 6cm in perpendicular dimensions, with patchy areas of contrast enhancement and coarse calcification (Figure 1D). The mass exerted substantial local mass-effect but with only minimal vasogenic edema. Pre-operatively, it was difficult to distinguish whether the mass was intra- or extra-axial. Gadolinium-enhanced MRI of the entire spine showed no evidence of additional lesions. No other masses were identified CT imaging of the body. A right parietal craniotomy for maximal safe surgical resection of the mass was performed urgently. Intra-operatively, all visible tumor was removed. The tumor appeared to be entirely extra-axial, without a defined site of origin in the brain parenchyma. There was no evidence of brain invasion and no distal dural deposits were observed. No residual tumor was identified on post-operative MR imaging studies. After extensive multidisciplinary discussion, treatment with concurrent radiation and temozolomide, as per the STUPP protocol, was initiated (1).

## Histology and molecular results

**Figure 2:**
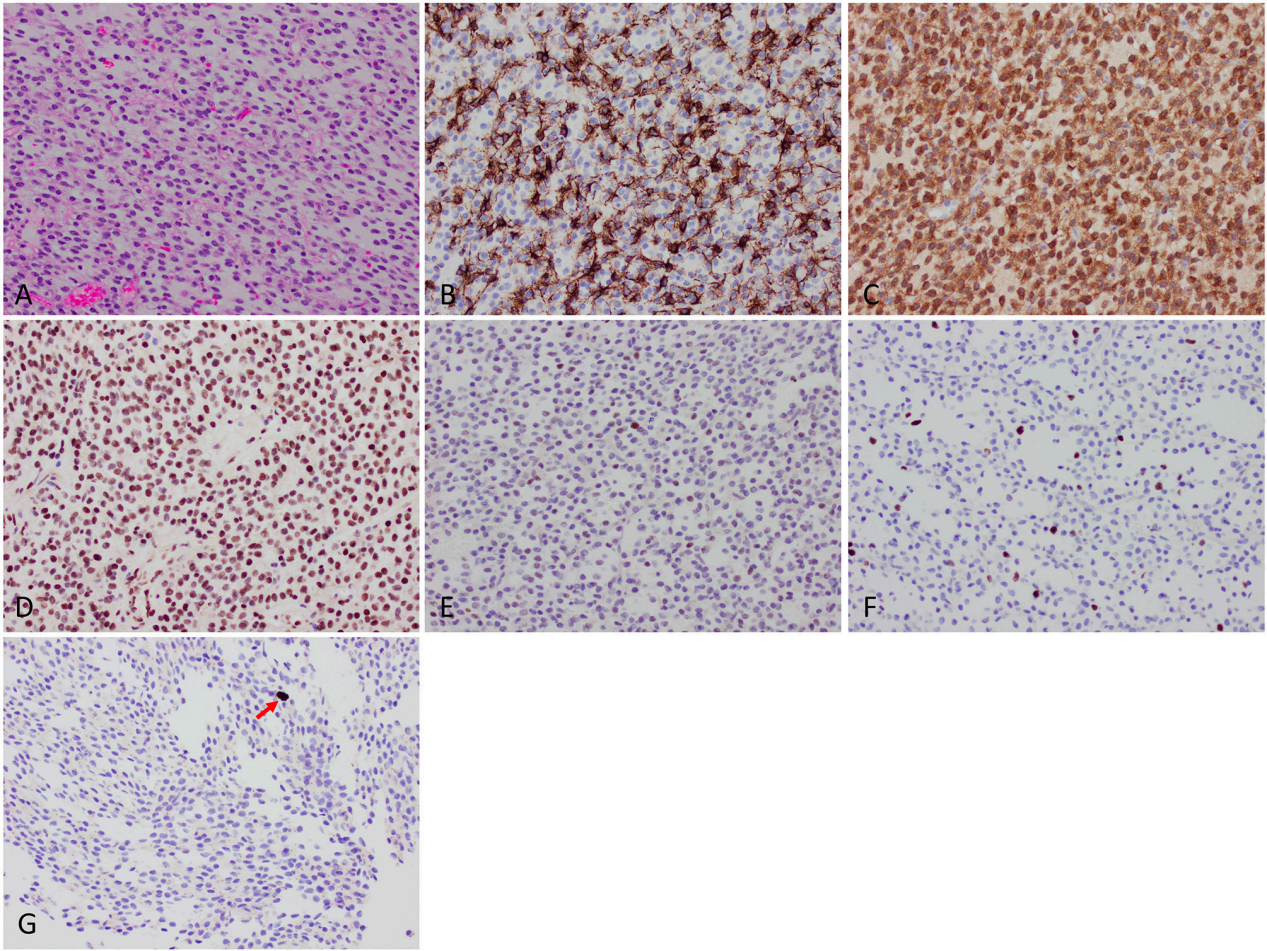
Histologic findings. **(A)** Microscopic examination showed diffuse sheets of a tumor composed of relatively small cells with round-to-oval nuclei and scant-to-cleared cytoplasm. **(B)** GFAP was positive in a subset of tumor cells. **(C)** IDH1 p.R132H mutant protein immunohistochemistry was strongly positive. **(D)** ATRX protein wildtype expression was retained. **(E)** Weak expression of p53 protein. **(F)** Low Ki67 labeling index of 4.8%. **(G)** Mitotic activity (arrow) was quantified at a maximum of 2 per 10 high-power fields using phosphohistone H3 (pHH3) immunostaining.

H&E-stained sections showed diffuse sheets of a tumor composed of relatively small cells with round-to-oval nuclei and scant-to-cleared cytoplasm (Figure 2). There were several small areas of stromal sclerosis with cell dropout, and collections of hemosiderin-laden macrophages were identified; in one of these foci, endothelial cell hypertrophy bordering on early hyperplasia was noted. No brain parenchyma was identified in the sections. Molecular signature and immunophenotype determination studies were performed. The tumor cells were immunopositive for GFAP, S100 protein, and mutant IDH1 p.R132H. ATRX immunostaining showed retained wildtype expression, with weak expression of p53 protein in a minor subset of tumor cells. Additional immunostains for synaptophysin, SMA, desmin, EMA, and keratins were negative. Mitotic figures were rare on H&E-stained sections, and were quantified at a maximum of 2 mitoses per 10 high-power fields using the phosphohistone H3 (PHH3) antibody. Computer-assisted quantitation yielded a correspondingly low Ki67 antigen (MIB1) labeling index of 4.8% (6,857 nuclei counted). Fluorescence *in situ* hybridization (FISH) studies showed a 1p/1q ratio of 0.58 and a 19q/19p ratio of 0.58, indicating the presence of 1p/19q codeletion in the tumor cells. Additional molecular testing (CARIS Molecular Intelligence, please visit https://www.carismolecularintelligence.com for a complete list of the genes evaluated) confirmed the presence of the *IDH1* c.395G>A p.R132H mutation and revealed a *CIC* c.601C>T p.R201W mutation (Table 1). The *MGMT* promoter (analyzed by pyrosequencing) was methylated. *ATRX* or *FUBP1* mutations and *BRAF-KIAA1549* fusion/tandem duplication at 7q34 were not detected. No mutations in the *BRAF* gene were identified. A final diagnosis of “Oligodendroglioma, IDH-mutant, 1p/19q-codeleted (leptomeningeal)” was rendered.

**Table 1:** Summary of genetic alterations.

**Gene/chromosome**	**Alteration**
*IDH1*	p.R132H
*ATRX*	Wildtype
*CIC*	p.R201W
*FUBP1*	Wildtype
*MGMT*	Methylated
1p	Deleted
19q	Deleted

## Discussion

In this case, the preoperative imaging studies showed an extra-axial mass with a small focus that appears inseparable from cortex (Figures 1A,B), raising the possibilities of either tumor pushing against the brain or a potential connection of the tumor to the brain parenchyma. The intraoperative observations of the neurosurgeon (solid tumor without an identifiable connection to brain parenchyma) and the results of the assessment of the resected tissue (as detailed above) indicate that this is an example of primary leptomeningeal oligodendroglioma (2–4). The other entity in the differential diagnosis is diffuse leptomeningeal glioneuronal tumor (DLGT), which very rarely can show combined 1p/19q codeletion (isolated 1p deletion is more common). IDH mutations have not been described in DLGT (2, 5–7). In contrast, DLGT or disseminated oligodendroglioma-like leptomeningeal neoplasms (DOLN) have been shown to frequently carry the *BRAF-KIAA1549* fusion/tandem duplication at 7q34 (6).

Review of the preoperative imaging studies demonstrated the presence of multiple prominent and unequivocal foci of ring-like contrast enhancement (Figure 1). A ring enhancement pattern is traditionally indicative of anaplastic changes (8). In this case, the tissue available for examination did not show frank vascular proliferation or necrosis; thus, the presence of unsampled WHO grade III tumor (anaplastic oligodendroglioma) is possible. However, recent studies indicate only a very modest, if any, prognostic impact of traditional histologic criteria-based grading for WHO grade II-III *IDH*-mutant diffuse gliomas, including oligodendrogliomas, with the most important prognostic factors being *IDH1/IDH2* mutation status and 1p/19q codeletion status (9, 10). CT imaging showed focal calcification (Figure 1D), supporting the clinical suspicion of the tumor's protracted natural history.

Several cases of primary leptomeningeal oligodendroglioma have been reported in the literature (4). An origin from meningeal glial heteroptopia has been postulated (3). However, many of the reported cases were not evaluated for 1p/19q codeletion or *IDH1/IDH2* mutation status, raising the question of whether the tumors represent true oligodendrogliomas, as defined in the 2016 WHO classification system. In contrast to primary leptomeningeal oligodendroglial tumors, involvement of the leptomeninges by parenchymal oligodendroglial tumors occurs only in a minority of patients (11). In this case, no parenchymal component was identified by imaging, intra-operative observation, or histologic studies, thus making this possibility unlikely.

In addition to *MGMT* promoter methylation, *IDH1* c.395G>A p.R132H mutation and 1p/19q codeletion, the tumor showed a *CIC* c.601C>T p.R201W mutation. Mutations in *CIC* are a frequent finding in oligodendroglioma (10). This case thus represents one of only a very few reports of molecularly-characterized, primary leptomeningeal oligodendroglioma in an adult patient. Primary leptomeningeal oligodendrogliomas are molecularly distinct from DLGT/DOLN, which are tumors that present in pediatric patients and frequently exhibit *BRAF-KIAA1549* fusion and 1p deletions. Although rare, primary leptomeningeal oligodendroglioma should be considered in the differential diagnosis of an extra-axial tumor with clear cell cytology. Testing for the critical molecular alterations (i.e., *IDH1/IDH2* mutations and 1p/19q codeletion) is essential for accurate diagnosis of this rare presentation of oligodendroglioma.

There is no standard evaluation and management for primary leptomeningeal oligodendroglioma. The authors suggest baseline staging of the CNS axis by imaging, and, if possible, by cerebrospinal fluid examination. There are no studies regarding long-term outcomes of adjuvant management in primary leptomeningeal oligodendroglioma comparing observation vs. adjuvant radiation with or without chemotherapy. This patient's age, health, preferences, and the possibility of microscopic disease involving the meninges and CSF, factored into the recommendation for adjuvant treatment. Given the presence of *MGMT* promoter methylation, as well as the accumulation of long-term results of several international prospective randomized clinical trials demonstrating improved outcomes with the addition of chemotherapy to radiation, the patient was commenced on radiation and temozolomide chemotherapy, as per the STUPP protocol (1). An alternative treatment strategy would have been the use of radiation followed by PCV (12). Long-term clinical-radiographic surveillance of the CNS is warranted.

## Ethics statement

This study was performed with approval of the institutional ethics committee (IRB) and with the patient's written informed consent for publication.

This study was carried out in accordance with the recommendations of the UT-MDACC IRB with written informed consent from all subjects.

## Author contributions

LB: Manuscript writing and figure preparation; ED: Clinical information and manuscript editing; NG-T: Figure preparation and manuscript editing; JH: Clinical information and manuscript editing; HC: Clinical information, specimen; JW: Specimen processing, slide review, manuscript editing; GF: Pathology and manuscript editing.

### Conflict of interest statement

The authors declare that the research was conducted in the absence of any commercial or financial relationships that could be construed as a potential conflict of interest.
